# Supplementary material for: Are carbon emissions trading and green financial instruments synergistic? -Comprehensive quantitative research based on content analysis
Source: PLoS One. 2024 Mar 7;19(3):e0298601. doi: 10.1371/journal.pone.0298601 (PMC10919601; doi:10.1371/journal.pone.0298601)
Supplement: S1 Data — (ZIP) [file pone.0298601.s001.zip › Data/地级市Z_F工作报告.zip!/┴╔─■/░░╔╜╩╨/└┤╫╘╣1⁄2╓┌║┼í╛┬φ┐╦╩2╛▌═°í┐_2015.docx]

各位代表：

现在，我代表市政府向大会报告工作，请予审议，并请市政协各位委员提出意见和建议。

一、2014年政府工作回顾

刚刚过去的一年，面对严峻复杂的经济形势，市政府在省委、省政府和市委正确领导下，在市人大、市政协监督支持下，紧紧依靠全市人民，牢牢把握“稳中求进”总基调，锐意进取、克难攻坚，采取一系列利当前、惠长远举措,较好完成了全年各项工作任务,主要指标增幅高于全省平均水平，经济运行基本保持在合理区间。预计，全年实现地区生产总值2721亿元，增长6%；公共财政预算收入241.7亿元,增长1%；固定资产投资1950亿元，增长5%；社会消费品零售总额899亿元，增长12.4%；城镇常住居民人均可支配收入28090元，增长9.5%；农村常住居民人均可支配收入12290元，增长12.5%；万元地区生产总值综合能耗下降4.2%。

（一）凝心聚力稳增长，经济运行总体平稳

全力帮企业保运行。把稳企业作为稳增长的重中之重。深入实施市政府领导包保重点企业责任制，为110户企业补办土地证、房产证，帮助企业解决各类难题237个。设立企业专项资金8.4亿元，发放工业企业转贷资金4.6亿元，减免、缓征各类涉企收费3.5亿元，通过“营改增”为企业减税3.8亿元。积极帮助企业开拓市场，组织企业参加国内外大型展销会57次，宝得等6户企业纳入全省工程采购目录。支持鞍钢调整产品结构，发展非钢产业，鞍钢等中省直企业为稳增长做出了突出贡献。支持民营企业平稳运行，后英集团等企业克服各种不利因素，做到税收不减、投资不降。全市工业用电量增长5.7%，列全省第1位，创10年来最好水平。

全力抓项目稳投资。把稳投资作为稳增长的压舱石。出台了《进一步促进投资增长的实施意见》,全面实施谋划提出、强力推进、开工建设、竣工投产、达标达产“五个百项工程”。实行市政府领导包保重点项目责任制，为138个项目解决土地、融资、配套等问题205个。丹佛斯供热设备制造基地、雨润物流交易中心等28个省级重点项目实现开复工，紫竹城市轨道钢、宏源激光等重点项目竣工投产，缘泰化工、瑞丰专用车、北京利尔、海华离子膜等重点项目加快建设。全年开工建设亿元以上项目954个，固定资产投资增幅列全省第2位。

全力抓招商增后劲。积极参加央企、民企、沪企“东北振兴辽宁行”。先后赴香港、台湾、广东、北京、上海、天津等地开展主题招商、产业链招商。与香港总商会等17个境外商协会和海外联行建立招商平台，与40多家世界500强企业、10多家央企构建伙伴关系。德国施奈尔、日本光触媒、海城卓达等176个重点项目签约落地和开工建设。全年实际利用外资15.8亿美元，增长14.5%；引进域外资金855亿元，增长20%。

全力抓机遇解难题。抢抓中央新一轮东北振兴机遇，按照国务院《关于支持东北振兴若干重大政策举措的意见》，分解148项重点任务，确定686个储备项目，落实责任逐项对接，铁西区成为国家首批城区老工业区搬迁改造试点，获得专项资金1.7亿元。抢抓国家棚户区改造政策机遇，争取国家棚改贷款资金57.5亿元，占全省争取资金总额的四分之一。不断拓宽融资渠道，鞍山城投公司成为交易商协会第一家市级融资平台。

全力防风险谋长远。严格落实国务院《关于加强地方政府性债务管理的意见》，严控债务规模，优化债务结构，提高使用效益，防范债务风险。债务融资成本降低3.5个百分点。

（二）坚定不移抓改革，发展活力有效释放

简政放权全面推进。把简政放权作为政府职能转变的突破口。出台《鞍山市政府转变职能简政放权实施意见》，在全省率先实施行政权力正面清单。按照“摸清底数、厘清边界、依法清理”原则，开展清权确权，对69个行政部门的9类行政权力进行全面审核清理，精简比例达38.9%。按照“能放就放、应放必放、放管结合”原则，向县（市）区下放68项市级管理权限，向开发园区下放47项市级管理权限。按照“事项公开、流程公开、要件公开”原则，对依法依规保留的318项行政权力全部面向社会公开。我市改革经验得到省委、省政府充分肯定并在全省推广。

审批制度改革全面深化。加强公共行政服务体系建设，市级行政审批事项全部进入公共行政服务中心集中办理，实行一个窗口受理、一站式审批、一条龙服务、限时办结。加强公共资源交易体系建设，市公共资源交易中心“管办分离”经验在《人民日报》显要位置刊发。

在全省率先完成工商营业执照、组织机构代码证、税务登记证“三证合一”改革试点。实行企业注册零门槛、零首付、零审查、零收费，全年新设公司制企业3204户，增长68.1%；新增企业注册资本226.4亿元，增长3倍。在全省首批进行消费投诉、经济违法行为举报、行政效能投诉“三台合一”改革试点，“12345”政府服务热线实现43个部门、87部公开投诉电话全覆盖。

政府机构改革有序进行。以提高行政效能为目标，在市场监管、农业农村、交通运输、城乡建设、卫生服务等领域实行“大部门制”改革，撤销、调整、理顺政府管理机构及具有行政职能的事业单位23个，政府工作部门由39个整合至35个。

（三）持续不断调结构，转型升级步伐加快

创新引领作用不断增强。坚持把科技创新作为产业升级的根本动力。组建高端装备、激光科技、高端阀门等研发检测公共服务平台。新增24家省级工程技术研究中心、重点实验室和产业专业技术创新平台。与哈工大、东北大学、大连理工等高校联合建设研究院，设立9个院士工作站。成立金融安全及系统装备等8个产业技术创新战略联盟。推进泰工机械等9个重点海外并购项目。激光产业园成为国家创新型产业集群，精细化工产业园成为国家火炬特色产业基地，海城菱镁新材料产业基地成为国家示范性国际科技合作基地，汤岗子新城成为省可持续发展示范区。全年新增国家级高新技术企业20家，总数达到100家；高新技术产品增加值占比提高到50%，增速列全省第2位。

农业现代化稳步发展。坚持用工业理念谋划农业。农业生产克服严重旱灾影响，实现粮食产量135万吨。水稻机插秧率达到90%以上，基本实现水稻全程机械化。海城南果梨、台安鸭鹅、岫岩食用菌等“一县一业”加快发展，实施投资10亿元的野生猕猴桃基地等农业产业化项目159个。加强农产品出口基地建设，岫岩成为国家级出口食用菌质量安全示范区，全年农产品出口增长54.6%。加强农村综合改革，海城成为全国农村改革试验区和新型城镇化试点市，在东北地区率先建立农村综合产权交易中心。

工业产业集群加速建设。坚持传统产业与新兴产业并重、重工业与轻工业并行。主导产业稳步增长，钢铁深加工、装备制造和菱镁新材料等产业增加值同比增长6.5%，鞍钢高强度汽车板、后英特种高纯镁砂等重点项目加速建设。新兴产业突飞猛进，激光科技、高端阀门、精细化工等产业投资成倍增长，增加值占比达到8.5%。激光产业园建成标准厂房50万平方米，注册企业194户，厦门弘信、武汉天琪等174个项目开工建设，俊达显示器、创鑫高功率激光器等80个项目竣工投产。轻工业发展迅速，中国现代软包装集团25万吨双向拉伸薄膜一期竣工投产，维达纸业6万吨高档生活用纸二期开工建设，青岛啤酒40万吨扩建项目加速推进。全年规模以上工业实现增加值1010亿元，增长6%。

现代服务业快速壮大。坚持把服务业作为转方式调结构的重要抓手。制定实施《加快服务业发展四年行动计划》，出台了8个重点行业扶持政策，实现了服务业用水、用电、用气与工业同价。服务业集聚区加快建设，14个服务业集聚区实现主营业务收入1350亿元，增长20%。海城专业市场集群晋升为全省示范服务业集聚区，投资60亿元的海城义乌中国小商品城等重点项目进展顺利。新兴服务业快速发展，新引进7家金融机构，新增金融机构本外币贷款271亿元，增长17.5%；新增中介服务业企业559家；完成74户企业生产性服务业分立，实现全年计划的137%；阿里巴巴产业带等4个电子商务平台加快建设，东北商品交易中心等4个大宗现货交易平台上线运营。旅游产业蓬勃发展，依托鞍山至北京、上海和新开通广州航线加大旅游宣传，千山荣获“2014最美中国旅游目的地景区”称号，全年接待国内外游客和旅游综合收入分别增长31%和39%。

（四）竭诚尽力惠民生，人民生活不断改善

重点民生工程取得突破。为城乡群众办的20件民生实事全面完成。将回迁房建设作为民生“一号工程”，多方筹措资金，建成启虹家园、丽水家园等10个项目185万平方米回迁房，1.9万户居民实现回迁。全年城镇实名制就业11万人，城镇登记失业率控制在2.59%。建立了统一的城乡居民基本养老保险制度，提高25万人新农保养老金待遇，企业退休职工养老金月人均增长177元，城镇35万名居民医疗保险实现大病统筹。城乡居民最低生活保障标准提高10%，资助904名困难家庭大学生就学。新建农村饮水工程82处，解决4.39万农民饮水安全问题，完成大伙房水源供水工程，新增日供水能力15万立方米，城市集中饮用水达标率100%。新购置清洁能源公交车200辆，新开通延伸公交线路6条，玉佛山绿道示范段建成使用。

基础设施加速建设。鞍山大道等47条道路、解放路立交桥等7座桥梁和48条城区街巷路新建改造工程全面完成，兴业环城大道全线通车，腾海大道主体竣工，通海大道、高铁大街等道路沿线绿化、美化、亮化、净化工程全面完成。维修养护农村公路150公里。新建改造供暖锅炉83台、管网204公里。鞍海一体化加速推进，汤岗子新城90公里“五横六纵”主干路网基本建成，核心区与景观节点初步形成。

生态环境持续改善。认真办理市人大关于加强大气污染治理“一号议案”，蓝天工程，投资46亿元，完成21座石灰竖窑永久性停炉、3台烧结机脱硫、4座气化竖窑改造、6座落后焦炉拆除、5台火电机组关闭等94个治理项目，利用鞍钢余热资源实施城市供热节能改造200万平方米。青山工程，完成荒山荒地造林6.35万亩，退坡地造林3.67万亩，墓园绿化3.69万株，矿山生态环境治理5759亩。碧水工程，已建成的12座污水处理厂全部投入使用，城镇污水处理率达到90%。净土工程，实施生活垃圾填埋场污染治理项目，解决了长期困扰周边居民的异味问题，实现了垃圾无害化处理率、覆盖率两个100%。全面启动宜居乡村建设，建成宜居示范镇10个、宜居示范村30个、宜居达标村324个。汤岗子新城荣获联合国环境署“全球绿色城市人居环境奖”。

社会事业全面发展。完成10所公办幼儿园、48所中小学和6所高中建设改造任务，新十三中学主体封顶；铁东区、立山区进入国家义务教育均衡发展示范县区行列；市职教城5所中职学校全部成为国家、省中等职业教育改革发展示范校。中国医大鞍山医院住院大楼开工建设，市妇儿医院儿科临床大楼主体工程完工，汤岗子新城公共卫生服务中心主体封顶；公立医院及基层医疗机构全部实行药品集中采购，市直及鞍钢所属医院药品加价率下降3个百分点；71所乡镇卫生院和22所社区卫生服务中心建设全面推进。新建12个基层图书馆、112个社区书屋和16个电子阅览室。完成岫岩山区农户“广播电视村村通”工程，实现了地区全覆盖。成功举办国际足球、篮球邀请赛，新购置群众体育健身器材1000余件。为30万名65周岁以上老人实施免费健康体检，为7800余名90岁以上老人发放高龄补贴1974万元；维修改造农村敬老院20所，新增民办养老机构19个。市殡仪管理服务中心主体封顶，市级公益性公墓主体工程基本建成。举行了首次烈士纪念日全市公祭活动。召开全市民族工作会议，对民族团结进步模范集体和个人进行表彰。创建全国文明城市的各项工作有序开展，城市文明程度和市民文明素质进一步提升。外事、侨务、对台、残联、统计、接待、史志、档案、气象、地震、人防、民兵预备役等工作取得了新成绩。

（五）真抓实干转作风，政府依法行政能力不断提高

民主法制建设不断加强。坚持依法行政，自觉接受市人大及其常委会法律监督、工作监督和市政协民主监督。全年办理人大代表建议、批评和意见197件、组合式议案1件，政协提案376件，见面率、办复率均为100%。制定和修正政府规章6件，受理行政复议案件30件。全年公开政府信息3.6万项，保证了广大群众的知情权、参与权、监督权。

社会管理水平不断提高。开展专项治理行动，集中整治机动车非法营运、三轮车无证经营、欺行霸市、占道经营等群众关注的热点问题。加强城市道路管理，增设千山路、鞍海路等交通安全隔离带，整顿站前中央商务区交通秩序。严厉打击各类违法犯罪，积极推进消防安全网格化管理，强化食品药品质量安全检查，全年未发生重特大生产安全事故和食品安全事件。切实做好信访工作，维护群众合法权益，社会保持和谐稳定。

机关工作作风明显改进。围绕为民务实清廉主题，聚焦“四风”问题，在政府系统扎实开展党的群众路线教育实践活动。结合政府工作实际，深入开展转变政府职能、工作方式和工作作风的“三转变、三提高”活动，集中整治各级政府机关不作为、乱作为等工作作风突出问题，服务群众的能力和水平实现了新提升。坚决落实中央八项规定、省市委十项规定，严格执行公务调研、公务接待、公务用车、办公用房、因公出国（境）等方面的制度规定，市本级“三公”经费支出下降6%。

各位代表，过去一年，面对经济下行压力，我们审时度势、奋发有为，实现了经济稳中有进、民生持续改善。这主要得益于市委的正确领导和市人大、市政协的鼎力支持，得益于广大干部压力之下不气馁、困难面前不低头的工作激情，得益于鞍钢等各类企业忘我拼搏、勇闯难关的突出贡献，更得益于全市上下凝心聚力、改革创新的共同奋斗。在此，我谨代表市政府，向为鞍山发展付出辛劳的各位代表和委员，向奋战在全市各条战线的广大干部和群众，向各民主党派、工商联、无党派人士、人民团体，向中省直单位、驻鞍部队、武警官兵，向关心支持鞍山发展的老领导、老同志，向所有为鞍山发展做出贡献的海内外朋友，表示衷心的感谢和崇高的敬意！

在肯定成绩的同时，我们更要清醒地看到经济社会发展中存在的矛盾和问题。一是产业结构不尽合理。资源型产业和重工业比重偏高，新兴产业和轻工业规模较小，现代服务业发展相对滞后。二是企业自主创新能力不强。产品科技含量较低，市场竞争力较弱，创新人才缺乏。三是环境压力和民生压力较大。生态环境改善和民生保障水平与群众期盼还有较大差距。四是政府职能转变步伐还需加快。简政放权等改革红利还没有完全释放，干部队伍中不作为、乱作为现象依然存在，依法行政水平有待进一步提高。这些都需要我们在今后的工作中采取有力措施，认真加以解决。

二、2015年主要工作任务

2015年是全面深化改革、全面推进依法治国的关键之年，是全面完成“十二五”规划的收官之年，也是深入实施新一轮东北振兴战略决策的开局之年，做好今年工作意义重大。

当前，我国经济发展进入新常态，经济运行正从高速增长转向中高速增长，经济发展方式正从规模速度型粗放增长转向质量效率型集约增长，经济结构正从增量扩能为主转向调整存量、做优增量并存的深度调整，经济发展动力正从传统要素投入转向以创新驱动为发展新引擎。我们要全面把握新常态呈现出的速度变化、结构优化、动力转换三大特点和阶段性特征，紧紧抓住中央新一轮东北振兴发展的战略机遇，清醒认识经过多年改革发展我们已经具备了雄厚的产业基础、良好的发展环境和强大的求发展、求振兴的发展合力。我们一定要坚定信心、保持定力、主动作为、乘势而上，努力开创鞍山老工业基地振兴发展的新局面。

2015年政府工作的总体思路是：全面贯彻党的十八大和十八届三中、四中全会精神，认真落实中央、省经济工作会议和市委十一届八次全会部署，牢牢把握“稳中求进”总基调，主动适应经济发展新常态，狠抓改革开放，突出创新驱动，加速转型升级，强化风险防控，加强民生保障，推进依法治市，全力推进经济社会平稳健康发展。

主要预期目标是：地区生产总值增长6%，公共财政预算收入增长2%，固定资产投资增长6%，社会消费品零售总额增长11%，城镇登记失业率控制在3%以内，确保城乡居民收入增幅高于经济发展速度。

（一）抢抓历史机遇，努力推进经济平稳健康发展

调速不减势，围绕新一轮东北振兴机遇，积极争取政策支持，稳定经济运行，集聚发展后劲，实现经济行稳致远。

抢抓振兴机遇。围绕破解难题抢抓机遇，力争在解决国企遗留问题、生态环境保护等重点领域先行先试，实现突破。围绕城市转型抢抓机遇，全力争取国家级资源型城市转型发展试点，推进立山区成为国家老工业区搬迁改造试点，争取大孤山、东鞍山、齐大山、海城镁矿、岫岩金矿等工矿区纳入国家独立工矿区搬迁改造试点。围绕促进发展抢抓机遇，谋划一批具有全局性、战略性、基础性的大项目，争取纳入国家支持范围。

支持企业发展。继续实施市级领导包保重点企业责任制，帮助企业解决实际困难，坚定企业发展信心。建立涉企收费清单制度，明确收费项目，规范收费行为，切实为企业“减负松绑”。加大政策宣传力度，积极鼓励现有企业扩大投资，享受招商引资同等政策，支持企业海内外并购做大做强。大力支持鞍钢等中省直企业发展，支持鞍钢矿山三期开发，围绕产业配套和外包服务，促进本地企业与鞍钢合作。支持民营企业发展，鼓励自主创新，提升市场竞争力。强化中小微企业金融服务，利用政府担保平台，扩大小额担保贷款覆盖范围。

推进项目建设。面向港台、日韩、珠三角、长三角、京津冀等重点区域，开展专业化招商、点对点招商、以商招商和平台招商，重点引进一批新兴产业、现代服务业和轻工业项目。建立招商引资跟进机制，切实做好洽谈、签约项目的跟进落实，力促一批招商项目落地实施。继续实施市级领导包保重点项目责任制，完善项目建设推进机制，做好土地整理、环评、基础设施配套、能源供给等全过程服务，确保项目早落地、早开工、早投产。全年开工项目1800个，其中亿元以上项目800个。

编制“十三五”规划。全面总结梳理“十二五”规划各项工作任务完成情况，深入开展调查研究，科学编制“十三五”发展总体规划以及各专项规划、区域规划，为鞍山描绘一张美好发展蓝图。建立产业动态、项目进展、环境治理等重点领域运行监测评价体系。

（二）深化改革开放，全面增强经济发展动力

坚持以改革为动力，做到“有效市场”和“有为政府”相结合，通过放宽市场准入，积极拓展对外开放的深度和广度，强力打造经济发展新引擎。

实施便利化改革。积极推进投融资体制改革，以便利化为目标，推进投资便利化、贸易便利化、金融便利化、商务便利化，建立权力平等、机会平等、规则平等的多元化投融资机制。探索负面清单制度，深化工商登记制度改革，变事前审批为事中事后监管，实行单一窗口受理，综合并联办理。实施权力清单动态调整。进一步向城区、开发园区下放市级管理权限，规范权力运行，优化审批流程，提高行政效能。

完善要素市场体系。扩大金融市场，鼓励民间资本设立金融机构，推进农村金融改革试点。支持鞍山银行股权转让、跨区经营。规划建设金融街，促进金融支持实体经济，鼓励企业中小板上市融资。建立健全城乡统一的建设用地市场，加大土地整理和腾净地工作力度，保证土地供应；扎实开展农村土地承包经营权确权登记颁证工作，引导农村土地有序流转。发展劳动力市场，围绕新兴产业、信息产业和劳动密集型产业，开展订单式职业技能培训，面向东北、内蒙、河北等周边省市引进多层次人力资源，满足产业发展需求。

放开投资领域。坚持“非禁即入”原则，放开公共服务、资源环境、生态建设、基础设施等重点领域的建设和运营市场。积极推广政府与社会资本合作（PPP）模式试点工作，引导社会资本投资燃气、供暖、教育、卫生、污水处理、垃圾焚烧等基础设施和公用事业项目，参与国有企业改制重组及合资经营。建立动态PPP项目库，规范选择合作伙伴。控制政府债务规模，优化政府债务结构，监管债务资金使用，严控债务风险。

实施“走出去”战略。充分发挥我市作为辽宁沿海经济带和沈阳经济区重要连接带的双重叠加优势，全面落实国家“一带一路”和京津冀协同发展战略，积极融入中蒙俄经济走廊建设，有效利用国际国内“两个市场”、“两种资源”。启动一次申报、一次查验、一次放行的“三个一”通关便利化模式，规划建设保税物流中心和保税仓库。重点扶持一批出口基地和龙头企业，推进我市先进装备、技术标准走出去，积极拓展多元化国际市场。

（三）坚持创新驱动，加速产业结构优化升级

坚持调整存量、做优增量并举，加快经济发展方式转变，优化产业结构，推进产业转型升级。

深入实施科技创新引领升级。鼓励全民创业、万众创新，激发科技创新活力。全面融入沈大国家自主创新示范区和沈大高新技术产业带，充分释放鞍山高新区发展潜能，建设国家自主创新示范区。全力推进鞍海城市带国家级蓝天科技工程可持续发展实验区建设。将“智慧鞍山”建设纳入全市中长期发展战略，积极申报“国家智慧试点城市”。搭建科技创新综合服务平台，建设国家知识产权示范市。强化企业技术创新主体地位，鼓励企业技术改造，扶持创新型企业发展壮大。加强科技人才队伍建设，广泛吸引一批科技创新、专业技能等高端人才来鞍创业发展。全年新设院士工作站5个，组建国家级、省级研发机构20家；新增国家高新技术企业20家，规模以上高新技术产品增加值增长10%。

深入推进工业“提质”升级。提高资源型产业发展质量和效益，进一步提升钢铁、菱镁等优势资源精深加工水平，提高产品附加值和资源综合利用率。加快推进激光科技、高端阀门、精细化工、健康制造等新兴产业集群建设。重点推进航天特种泵阀产业园、中科化工煤焦油、海龙重工机械产业园等项目建设。落实促进激光产业发展30条政策，设立5亿元激光产业发展资金，加速发展光电信息、四代移动、终端触屏等产品，引进产业配套企业，打造激光科技全产业链；全面建成光通信园、电源园、哈工大工业园等园区，力争全年入驻企业80家。大力发展轻工业，加速推进台安安井、鞍山元首、青岛啤酒等重点项目开工建设、竣工达产。全年新增规模以上工业企业50家，规模以上工业增加值增长6%。

深入推进服务业“提速”升级。加速传统服务业向专业化、体验式升级，重点发展服务业集聚区、专业化大市场和集购物、餐饮、住宿、休闲、娱乐为一体的体验式消费，加快推进万熹城市广场、上海城、YOYOMA-LL等项目建设。加速新兴服务业向金融证券、电子商务等领域拓展，重点发展网络交易、线上运营等电子商务平台，引进基金、金融租赁公司，推进多层次资本市场建设；支持阿里巴巴产业带、大德电商谷创建省级示范园区。生产性服务业向技术研发、配套流通和再制造服务升级，重点发展设备租赁、仓储物流、研发设计等生产服务，积极推进辽宁钢铁物流基地、万贯国际工业品博览中心等项目建设。发展体育产业，出台促进体育消费政策，筹建乒乓球学校等集训基地，积极承办大型体育赛事。加速推进中国（鞍山）数字娱乐电子竞技产业园等项目建设。推进棚户区、城中村、城边村搬迁改造，鼓励货币化安置，办好春夏秋房交会，有效释放住房刚性需求，促进房地产业健康有序发展。

深入推进农业“提效”升级。加快推进农业设施化，重点发展设施种植业、设施养殖业、设施林业和设施渔业。加快推进农业产业化，加速海城、台安、岫岩农高区建设，重点推进野生猕猴桃基地、高端水稻生产基地等项目建设，全年新培育规模以上农事龙头企业10家。转变农业生产经营方式，加快推进农业规模化，重点发展三星、聚龙、聚源等经营主体，农民专业合作社发展到2500个，家庭农场发展到220家。

深入推进文化旅游产业“提档”升级。把发展文化旅游产业作为调结构的主攻方向。召开全市旅游产业发展大会，加大政府投入，促进旅游产业发展。深度挖掘千山、玉佛、温泉、冶铁等旅游资源历史文化内涵，开发工业遗产、美丽田园观光等旅游项目。完善城市游客服务中心、旅游标识系统等旅游设施。加快旅游项目开发建设，重点推进千山国际文化旅游产业园和传统文化展示演艺中心、海城牛庄古城、辽河台安生态旅游区、岫岩药山、千山大熊猫野生动物园等项目建设。加大旅游宣传力度，利用电视、报纸等传统媒体和网络、微信等新兴媒体，实施全方位、多层次、立体式的旅游宣传，拓展旅游市场空间。打造旅游品牌，推出“大东北五城市”精品旅游线路，办好千山国际文化旅游节和南果梨文化旅游节，推进千山、玉佛山创建国家5A级旅游景区，努力打造具有国际影响力的旅游目的地城市。全年接待国内外游客和旅游总收入双增长25%以上。

（四）统筹城乡建设，全面打造宜居宜业人居环境

坚持规划为引领，统筹推进城乡建设，加快生态文明建设，着重提升发展质量，切实改善城乡群众生活环境。

加速推进鞍海一体化。按照“以人为本、四化同步、优化布局、生态文明、文化传承”原则，加快镇村建设规划修编，科学规划新型城镇化布局，积极稳妥推进新型城镇化建设，构建新型城镇化体系。以汤岗子等新城新市镇建设为重点，加快推进建设大道、黄山路、鞍山路东西延等道路建设，加快推进350健康产业园、鞍山塔、文化中心和规划中心等项目建设，把汤岗子新城打造成为全国循环产业示范基地。实现鞍海交通一体化、基础设施一体化、产业一体化。

加快城乡基础设施建设。全面推进民生西路大修、千山中路拓宽、五一路立交桥维修等46项道路桥涵工程，兴业环城大道二期绿化亮化等16项“四化”工程，达道湾停车场等8项公共交通工程，以及弘扬路排水、东环路燃气配套、园林地区供热管网等34项管网改造工程。改扩建机场航站楼，谋划西南、西北地区航线。修建行人过街天桥5座，改造城乡公交候车亭686组，加快建设营城子公园、万水河公园。推进海岫铁路提级改造。建成500千伏长岭输变电工程和220千伏联网工程。维修改造100公里村级公路。加快宜居乡村建设，推进村屯基础设施改造、垃圾处理等综合整治，确保年内建成15个宜居示范镇、60个宜居示范村、700个宜居达标村。

加强生态环境建设。编制实施《城市环境总体规划》和《河流城市段滨水生态景观规划》。蓝天工程，大力推进燃煤锅炉除尘改造，完成鞍钢炼钢三工区整体改造等一批重点工程项目，关闭北美第二热电厂，加大矿山扬尘、建筑扬尘、道路遗撒、秸秆焚烧等综合整治力度；积极推进“气化鞍山”，加快建设天然气母站和输气管网；新增200辆清洁能源公交车，实施100辆公交车油改气；继续推进利用鞍钢余热资源实施城市供热节能改造600万平方米。青山工程，按照“谁开发谁保护，谁破坏谁恢复”原则，扎实推进矿山生态环境治理，全年完成荒山荒地造林3.7万亩，墓园绿化1.43万株，矿山生态环境治理2000亩。碧水工程，建成永宁污水处理厂，加快建设汤岗子和台安工业园区污水处理厂，城市污水收集率达到100%，结束城区污水直排历史。净土工程，加快循环经济产业园建设，开工建设生活垃圾焚烧发电、污泥处理等5个项目。

（五）切实改善民生，让发展成果更多更公平惠及人民群众

树立政府过紧日子，群众过幸福日子理念，把更多的物力、财力用在保障和改善民生上。切实做好20件民生实事。

继续实施民生工程。全力加快回迁房建设，确保建成友谊家园、四达花园等11个项目138万平方米回迁房，保证1.6万户动迁居民按时回迁；通过货币化安置和政府团购方式，进一步解决长期未能回迁居民的安置问题。坚持以创业带动就业，加强技能培训和创业培训，完善创业服务体系，对就业困难家庭实施100%就业援助；为城乡妇女创业提供小额担保贷款财政贴息。完善社会保障体系，加速推进“五险合一”系统建设，提高职工医保和居民医保门诊统筹报销标准，新型农村合作医疗筹资标准提高到460元，免费发放城市居民健康卡和新农合居民健康卡，城乡最低生活保障标准提高7%以上。

加快发展社会事业。新建改造15所幼儿园、20所中小学。新建改扩建五中教学楼和三中、九中体育馆。推进宽带网络校校通、优质资源班班通和网络学习空间人人通，完善教育资源和教育管理公共服务平台，全面实现基础教育信息化。加快青少年科技实训基地建设。促进职教城成为国家职业教育综合改革试点，争创省职业教育示范园区。支持辽宁科技大学发展，加快鞍山师范学院新校区建设。深化公立医院改革，积极推行药品零加价，推动医疗服务价格机制、医保支付机制和医院管理体制综合配套改革。加强医院管理，引进优质医疗资源和人才，切实提高医疗服务能力。加快推进市中心医院全科医生培养基地建设。继续创办市图书馆分馆和流动图书馆，将150个社区书屋纳入公共图书馆“一卡通”系统。完成市博物馆升级改造工作。举办鞍山市第十届运动会。广泛开展全民健身活动，新建100个城乡健身广场，20所中小学体育设施分时段向社会开放。发展社会养老事业，完善城乡统一的居民基本养老保险制度，强化具有日间照料和居家养老功能的养老服务设施，建成海城、台安、岫岩社会福利中心。继续推进市殡仪管理服务中心和市公益性公墓建设。积极做好双拥模范城创建工作。

不断加强社会治理。深入推进平安鞍山、法治鞍山建设。完善社会治安立体防控体系，依法严厉打击各类违法犯罪活动，不断提升人民群众的安全感和满意度。加强安全生产和交通消防安全隐患排查治理，坚决遏制重特大安全事故发生。强化食品药品安全监管，从源头做起，建立覆盖生产、流通、消费各环节的食品药品安全监管制度，严厉打击生产销售假冒伪劣食品药品行为，保证群众“舌尖上的安全”。完善城市管理体系长效机制建设，持续开展市容环境综合整治。加大信访问题排查化解力度，创造和谐稳定社会环境。

进一步做好妇女儿童、老龄、残联、民族、宗教、外事、侨务、对台、统计、接待、史志、档案、气象、地震、人防、民兵预备役等工作。

（六）坚持依法行政，打造人民满意政府

按照为民务实清廉要求，切实加强作风建设，努力打造法治型、责任型、服务型、廉洁型政府，不断提升政府的执行力和公信力。

建设法治政府。把遵守宪法和法律作为政府工作的根本原则。依法接受市人大及其常委会法律监督、工作监督和市政协民主监督，办好人大代表议案、建议和政协提案。政府重大决策向人大及其常委会报告，向政协征求意见，广泛听取各民主党派、工商联、无党派人士意见和建议。认真贯彻落实《中共鞍山市委关于全面推进依法治市的实施意见》。落实备案审查条例，清理政府规章和规范性文件。全面推进政府信息公开，加大财政预算、公共资源配置等信息公开力度，自觉接受群众监督、舆论监督和社会监督。

建设责任政府。巩固扩大教育实践活动成果，切实抓好整改落实、建章立制各项工作。坚持实事求是、求真务实，对政府出台的政策，对企业、群众和投资者作出的承诺，坚决落实到位，做到言必信、行必果，切实增强政府公信力。大力倡导勇于担当、敢于负责的精神，坚持打基础、利长远，一张蓝图画到底，狠抓工作落实，明确责任清单、时间进度表和检查标准，定领导、定责任、定措施、定期限，确保各项工作落实到位。

建设服务政府。牢固树立亲民为民理念，努力办好顺民意、解民忧、惠民生的好事实事。牢固树立服务企业理念，认真解决好事关企业发展的难事急事。充分发挥公共行政服务中心、公共资源交易中心和“12345”政府服务热线等服务平台作用，努力提高公共服务水平。

建设廉洁政府。加强反腐倡廉制度建设，强化重点领域、关键环节的行政监察和审计监督。切实把艰苦奋斗、厉行节约内化到政府运行各环节，严控“三公”经费支出，全面完成公车改革。深入整治机关作风，持续治理庸懒散奢、不作为乱作为现象，切实转变工作作风，努力打造一支锐意进取、勤政为民、清正廉洁的公务员队伍。

各位代表、同志们，让我们以党的十八届三中、四中全会精神为指引，在省委、省政府和市委的正确领导下，在市人大、市政协监督支持下，抢抓机遇，锐意改革，敢于担当，主动作为，适应新常态，开创新局面，为实现鞍山老工业基地全面振兴而努力奋斗！
